# Mechanistic Aspects of [3+2] Cycloaddition Reaction of Trifluoroacetonitrile with Diarylnitrilimines in Light of Molecular Electron Density Theory Quantum Chemical Study

**DOI:** 10.3390/molecules30010085

**Published:** 2024-12-29

**Authors:** Agnieszka Łapczuk, Mar Ríos-Gutiérrez

**Affiliations:** 1 Department of Organic Chemistry and Technology, Cracow University of Technology, Warszawska 24, 31-155 Cracow, Poland; 2 Department of Organic Chemistry, University of Valencia, Dr. Moliner 50, Burjassot, 46100 Valencia, Spain

**Keywords:** [3+2] cycloaddition, nitrilimine, reactivity, molecular mechanism, molecular electron density theory, 1,2,4-triazole

## Abstract

In this study, we investigated the [3+2] cycloaddition reaction of CF_3_CN (TFAN) with nitrilimine (NI) to produce 1,2,4-triazole and compared the resulting isomers. We determined the preferred reaction pathway by examining the electrophilic and nucleophilic properties of the reaction substrates, performing thermodynamic calculations for the individual pathways, and comparing them with the experimental results.

## 1. Introduction

**Scheme 1:**
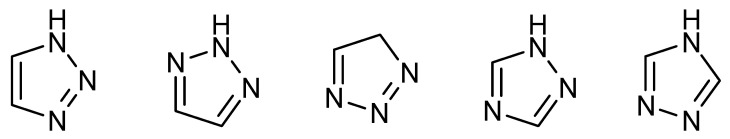
Structures of 1,2,3- and 1,2,4-triazoles.

There has been a rapid development of heterocyclic chemistry in the context of pharmacology [1]. Among the range of compounds that deserve special attention are triazoles (Scheme 1). These compounds are widely used as drugs due to their strong bioactivity. Organic compounds containing a triazole scaffold as a core unit, especially 1,2,4-izomers [2,3,4,5], possess antifungal [6], antibacterial [7,8], antitubercular [9], antiviral [10,11,12], anti-inflammatory [13], anticancer [14], antidepressant [15] and analgesic activities.

A common practice in synthetic chemistry involves the modification of the triazole ring by adding substituent groups, which can significantly enhance the biological activity of this class of organic compounds [16]. Of particular interest in this context are the trifluoromethyl-substituted triazole derivatives, which have garnered attention due to their promising pharmacological properties. The introduction of the trifluoromethyl group skeleton further enhances these properties and paves the way for their application in even more effective medications [17,18,19,20]. The inclusion of fluorine atoms in the molecule can influence the rate of drug metabolism, improve the stability of drug metabolism [21] and enhance the biological [17,22], antibacterial and anti-HIV [23] activity.

Despite such intriguing properties, current synthetic approaches for trifluoromethylated 1,2,4-triazoles are scarce and suffer from some limitations. The most common and popular problems include utilizing transition metal catalysis, the use of the explosiveness of diazo and diazonium reagents, the complexity of the reaction mixture, and poor regioselectivities [24]. The synthesis of such compounds is an intensively researched and rapidly evolving topic in the scientific literature [25,26,27,28,29,30,31].

**Scheme 2:**
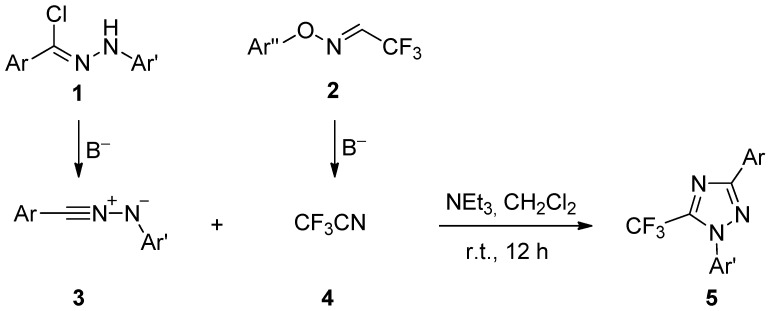
Experimental 32CA reaction of NI **3** with TFAN **4**, yielding triazoles **5**.

Generally, the nitrile bond is characterized by very low activity in cycloaddition reactions. Very rare cases of non-catalyzed reactions from this group regard the cycloaddition between aromatic nitrile *N*-oxides and cyano-substituted nitroethenes [32]. You and Weng et al. recently reported a general procedure [33] to obtain 5-trifluoromethyl 1,2,4-triazoles via the [3+2] cycloaddition (32CA) reaction of nitrileimines (NI) **3**, generated in situ from hydrazonyl chloride **1**, with trifluoroacetonitrile (TFAN) **4**, generated from the precursor 2,2,2-trifluoroacetaldehyde *O*-(aryl)oxime **2** (see Scheme 2). The reaction was carried out at room temperature for 12 h in dichloromethane solution, yielding 1,3-diaryl 5-trifluoromethyl triazoles **5** as a single product with good yields (39–98%). The authors found that the variable yields stem from the significant effect of NI’s aryl substituents on the reaction efficiency. While strong electron-donating groups (EDGs) favor the reaction, strong electron-withdrawing groups (EWGs) furnish moderate yields, and halogen substitutions provide good yields. It is interesting that in analogous 32CA, with the participation of diarylnitrilimines and a CCl_3_-substituted molecular system, the HCCl_3_ molecule is spontaneously eliminated from the primary cycloadduct [34]. This underlines the special role of the perfluoroalkyl substituent within the reaction course.

Molecular Electron Density Theory (MEDT) [35] provides a modern interpretative framework to understand reactivity in 32CA reactions. Recent MEDT studies have established a very good correlation between the electronic structure and the reactivity of the three-atom components (TACs) participating in 32CA reactions. Accordingly, 32CA reactions have been classified into four different types, namely, pseudodiradical (pdr), pseudo(mono)radical (pmr), carbenoid (cb), and zwitterionic (zw). While the pdr-type reactions of pseudodiradical TACs, such as azomethine ylides, present very high reaction rates even with a low polar character, the zw-type reactions of zwitterionic TACs, such as nitrones, demand suitable nucleophilic/electrophilic activation to take place easily.

**Scheme 3:**
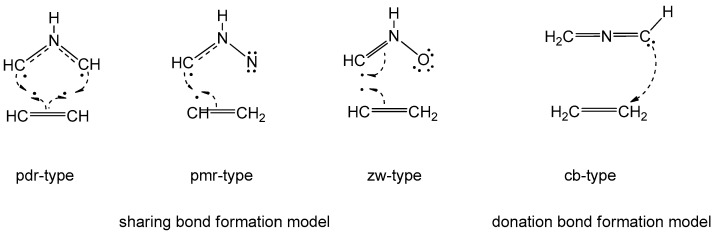
Sharing and donation bond formation models.

The simplest NI is a carbenoid TAC, which participates in cb-type 32CA reactions [36]. The same as the other three types, these reactions demand adequate substitution but proceed through a different mechanism. While pdr-, pmr- and zw-type reactions involve the sharing bond formation model via pseudoradical centers, the presence of a carbenoid center in the simplest NI implies a donation bond formation model [37] (see Scheme 3). However, substitution can change the electronic structure of TACs and, therefore, the corresponding molecular mechanism and reactivity.

**Scheme 4:**
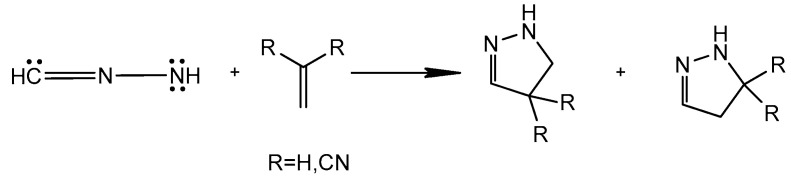
32CA reaction of NI with ethylene and dicyanoethylene (DCE).

Among the four types of 32CA reactions, the cb-type reactions have been the least theoretically studied within MEDT due to a scarcer number of cases. Only nitrile ylides (NYs) have been characterized as carbenoid TACs, and their reactivity has been fully established [38]. However, in the case of NIs, only the simplest 32CA reaction of NIs with ethylene and dicyanoethylene (DCE) has been studied. That study showed an ortho regioselectivity in the reaction with DCE (see Scheme 4).

On the other hand, the effect of CF_3_ substitution has been previously studied. It was found that this EWG enhances the electrophilic character of the species, causing no significant changes in the local electrophilic activation.

The purpose of the present work is to provide an explanation for the total selectivity experimentally observed in the 32CA reactions of 1,3-diaryl NIs with *trifluoroacetonitrile* (TFAN). To do so, a 32CA reaction involving the simplest experimental *C*-aryl-*N*-phenyl NI **3a**–**c**, yielding 1,3-diphenyl 5-trifluoromethyl 1,2,4-triazole **5a**–**c**, was used as the computational reference. Then, two substituted aryl triazoles were used to explain the dependence of the reactivity of the corresponding NI with the electronic nature of the substituents. Several quantum chemical tools were used to characterize the reactivity and electronic structure of the reagents, as well as explain the mechanism and the factors responsible for the reaction rates of the reactions involving experimental NIs.

## 2. Results

The present theoretical study is divided into six parts: (i) first, a topological analysis of the electronic structure of NIs **3a**–**c** and TFAN is performed in order to classify the NIs into one of the four TAC structures; (ii) next, the reactivity indices at the ground state of the reagents are analyzed in order to predict the reactivity and regioselectivity in these 32CA reactions; (iii) then, the energy profiles associated with the 32CA reactions of NIs **3a**–**c** with TFAN are studied; (iv) this is followed by a quantum topological study of the bonding changes along the most favorable reaction path of the 32CA reaction between *C*-aryl-*N*-phenyl NI **3a**–**c** and TFAN in order to characterize the molecular mechanism of this cycloaddition; and (v) finally, the origin of the activation energy of these reactions are studied by applying a real-space quantum topological energy partitioning scheme.

### 2.1. Analysis of the Electronic Structures of the Reagents

**Figure 1:**
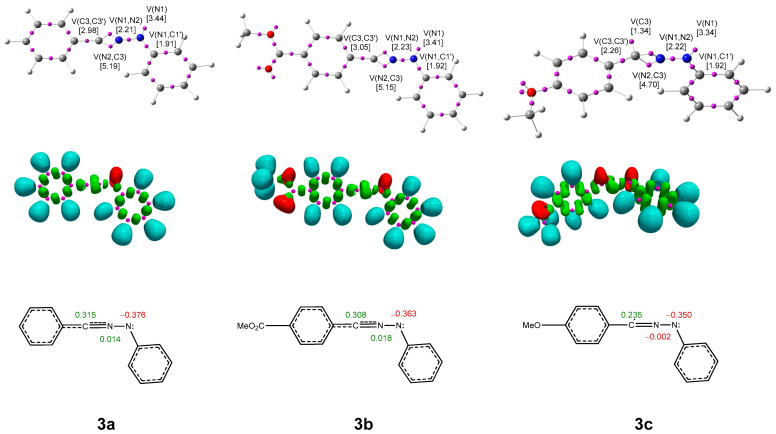
ωB97X-D/6-311g(d,p): ELF localization domains of TAC represented at an isosurface value of ELF = 0.75 on the top side. ELF basin attractor positions, together with the most representative valence basin populations, in the center and the proposed ELF-based Lewis structures, together with the natural atomic charges, on the bottom side. Negative charges are colored in red, and positive charges in green. ELF valence basin populations and natural atomic charges are given as average number of electrons (e).

**Figure 2:**
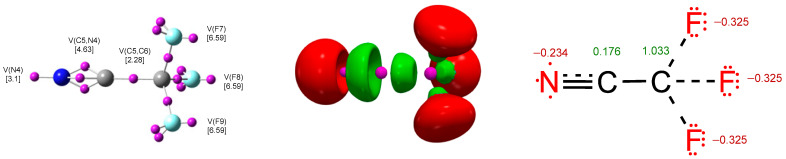
ωB97X-D/6-311g(d,p): ELF localization domains of TFAN represented at an isosurface value of ELF = 0.75 on the top side. ELF basin attractor positions, together with the most representative valence basin populations, in the center, and the proposed ELF-based Lewis structures, together with the natural atomic charges, on the bottom side. Negative charges are colored in red, and positive charges in green. ELF valence basin populations and natural atomic charges are given as average number of electrons.

The electron localization function (ELF) [39] is a valuable tool for both quantitatively and qualitatively describing the electronic structure of organic molecules. Thus, taking into account the established structure–reactivity relationships for TACs, an ELF topological analysis of aryl NIs **3a**–**c** and TFAN **4** was performed in order to characterize their electronic structure and potentially predict their reactivity in 32CA reactions. Figure 1 displays the ELF localization domains and the ELF basin attractor positions, together with the most relevant valence basin populations (Table 1), as well as the corresponding proposed Lewis-like structures for NIs **3a**–**c**, while Figure 2 shows the same data for TFAN **4**.

The ELF topological analysis of NI (**3a**) shows the presence of two disynaptic basins within the N2–C3 bonding region, V (N2–C3) and V′ (N2–C3), involving a total population of 5.19e. This indicates that the N2–C3 bond possesses a triplet-bond character. The N1-C1′ and C3–C3′ bonding regions in NI (the bond with the phenyls groups) are characterized by the presence of V (N1, C1′) and V (C3, C3′) disynaptic basins, with a population of 1.91e and 2.98e, respectively. It shows the presence of V (N1) monosynaptic basins integrating a total electron population of 3.44e and one V (N1, N2) disynaptic basin integrating an electron population of 2.21e. Upon inclusion of the electron-withdrawing carboxylate group –CO_2_Me at the para position of the phenyl substituent (**3b**), no significant changes were observed in the results of the ELF topological analysis. On the other hand, substitution with the electron-donating methoxy group –OMe (**3c**) leads to notable alterations in the C3–C3′ bonding regions. The V (C3, C3′) disynaptic basin, with an electron population of 2.26e, is less electron-rich compared to **3a** and **3b**, which results in the bond acquiring a more single-bond character. Additionally, a V (C3) monosynaptic basin with an electron population of 1.34e appears.

Topological analysis of the ELF shows no carbenoid or pseudoradical centers at NI **3a** and **3b** but one carbenoid center at **3c** with the inclusion of the methoxy –OMe group. This means that the electronic structure of NI **3** is prone to change with substitution, making **3c** a carbenoid TAC participating in cb-type 32CA reactions.

The natural atomic charges of the most relevant centers of **NI 3a**–**c** are shown together with the proposed ELF-based Lewis structures given in Figure 1. The N1 nitrogen center is negatively charged by ca. 3.335e, and the N2 and C3 centers present charges of −0.146e and 0.284e, respectively. These values, as well as the computed dipolar moments of 4.26D, indicate that these NIs present a charge separation that makes them dipolar species but in contrast with the expected charges arising from Lewis’s bonding model (see Figure 1). Note that the molecular charge distribution is the consequence of the asymmetric electron density delocalization within a molecule, resulting from the presence of different nuclei in the molecule, rather than the consequence of the resonance Lewis structures.

On the other hand, the ELF topological analysis of TFAN (**4**) shows the presence of three disynaptic basins within the N4-C5 bonding region, V (N2–C3), involving a total population of 4.63e. This observation may suggest the existence of an overpopulated double bond. A singular covalent linkage is evident between carbon atoms C5 and C6, with the concomitant observation of a disynaptic basin denoted as V (C5, C6), integrating a total electron population of 2.28e. This analysis also reveals the existence of three monosynaptic basins, each incorporating a total electron density of 5.59e at the respective fluorine atoms F7-9. Additionally, a monosynaptic basin can be discerned at the nitrogen atom N4, with an electron population of 3.41e.

Figure 2 illustrates the natural atomic charges of the most relevant centers and the proposed ELF-based Lewis structure. Negative charges are primarily localized on the three fluorine atoms (−0.325e each), while a negative charge (−0.234e) is found on the nitrogen atom (N4) within the nitrile group.

### 2.2. Analysis of the Reactivity Indices of the Reagents

Analysis of the reactivity indices defined within the CDFT [40,41,42] is a valuable instrument for comprehending the reactivity of organic molecules participating in polar reactions. Using this method, it is possible to easily assign each addend the role of either a nucleophile or an electrophile in the reaction being studied [42,43]. The global indices, namely, the electronic chemical potential (µ), chemical hardness (ƞ), electrophilicity (ω) and nucleophilicity (N), for NI **3a**–**c** and TFAN **4** are presented in Table 2.

**Figure 3:**
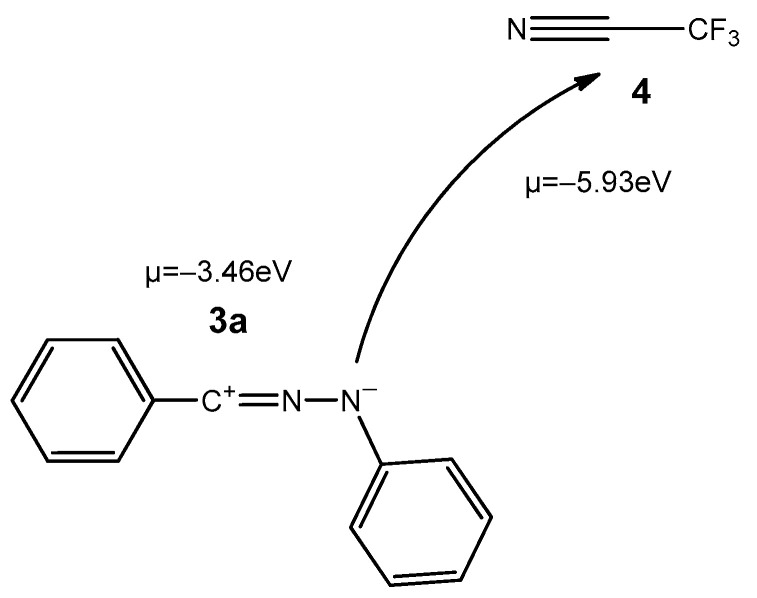
Visualization of the flux of the electron density between trifluoroacetonitrile **4** and C,N-diphenylnitrilimine **3a**.

In the case of the reaction between trifluoroacetonitrile (**TFAN**) 4 and C,N-diphenylnitrilimine (**NI**) **3a**, the electronic chemical potentials μ [35,44] of trifluoroacetonitrile (TFAN) **4** and C,N-diphenylnitrilimine (NI) **3a** are equal at −5.93 eV and −3.46 eV, respectively (Table 2). This indicates that in the course of this reaction, the global electron density transfer (GEDT) will take place from nitrilimine **3a** to trifluoroacetonitrile **4** (Figure 3). The chemical potential value for substituted derivatives of NI does not undergo significant changes when an aryl group is replaced with the CO_2_Me (**3b**) or OMe (**3c**) groups at the 1,4 positions relative to the carbon atom C3. The respective chemical potential values are −3.86 eV and 3.43 eV. Thus, the direction of electron flow between molecules **3a**–**c** and **4** remains unchanged, with all of them being classified as forward electron density flux (FEDF) reactions [45].

The chemical hardness η values for the substituted derivatives of TAC **3a**–**c** are similar and independent of the substituent, ranging from 6.51 to 7.59 eV. Conversely, this indicator for molecule **4** is significantly higher, measuring 14.25 eV, which indicates a higher resistance to electron density changes.

The electrophilicity ω [46] and nucleophilicity N **[47]** indices of NI **3a** are ω = 0.86 eV and N = 4.85 eV. According to the scales proposed by Domingo and co-workers [42], this compound should be treated as a moderate electrophile in a polar reaction due to the electrophilicity scale [40,48]. Substituted NI derivatives also exhibit supernucleophilic behavior. TFAN’s global indices of electrophilicity at ω = 1.23 and nucleophilicity at N = −1.25 classify this compound as a strong electrophile and marginal nucleophile.

**Figure 4:**
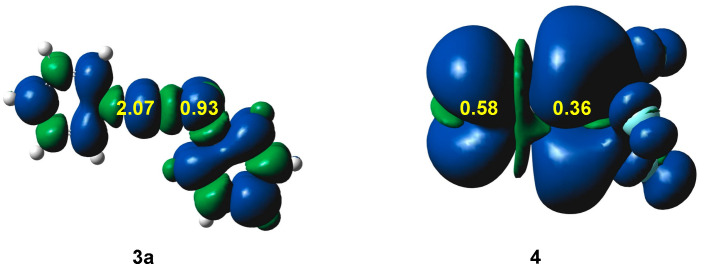
Three-dimensional representations of the Mulliken atomic spin densities of C,N-diphenylnitrilimine **3a** and trifluoroacetonitrile **4**, together with the nucleophilic Pk^−^ and the electrophilic Pk^+^ Parr functions of **3a** and **4**, respectively.

Identifying the role of nucleophiles and electrophiles in the studied reactions is particularly useful for reactions where the formation of multiple isomeric products is possible [49,50]. In order to ultimately determine the most preferred reaction pathway from a theoretical perspective, it is essential to analyze the local electronic properties of the atoms involved in the formation of new bonds in the structure of the resulting ring [49,51]. The analysis of the nucleophilic Parr functions (Pk^−^) at the reactive sites of NI **3a** reveals that the N1 nitrogen, with a Pk^−^ value of approximately 2.07, is the most nucleophilic center of this species. In contrast, the C3 carbon is only half as nucleophilically activated as the N1 nitrogen (0.93). On the other hand, examination of the electrophilic Parr functions (Pk^+^) at the reactive sites of TFAN **4** shows that the C5 carbon has the most electrophilic center, with a Pk^+^ value of 0.58 (see Figure 4). Consequently, it can be predicted that the most favorable nucleophilic/electrophilic interaction in the polar process of the attack of NI **3a** on TFAN will occur between the N1 nitrogen center, the most nucleophilic site of NI, and the C5 carbon, the most electrophilic site of TFAN. A very similar situation is expected in the case of NI **3b**, where the N1 nitrogen (Pk^−^ = 1.96) will interact with the C5 carbon in TFAN. However, it is noteworthy that the -OMe group in **3c** alters this scenario, as the most nucleophilic center becomes the C3 carbon, seemingly due to the presence of the C3 pseudoradical center. Even so, the values for both atoms are quite similar (Pk^−^ = 1.96 and 1.58, respectively).

### 2.3. Study of the 32CA Reaction Between NI ***3a–c*** and TFAN 4

**Scheme 5:**
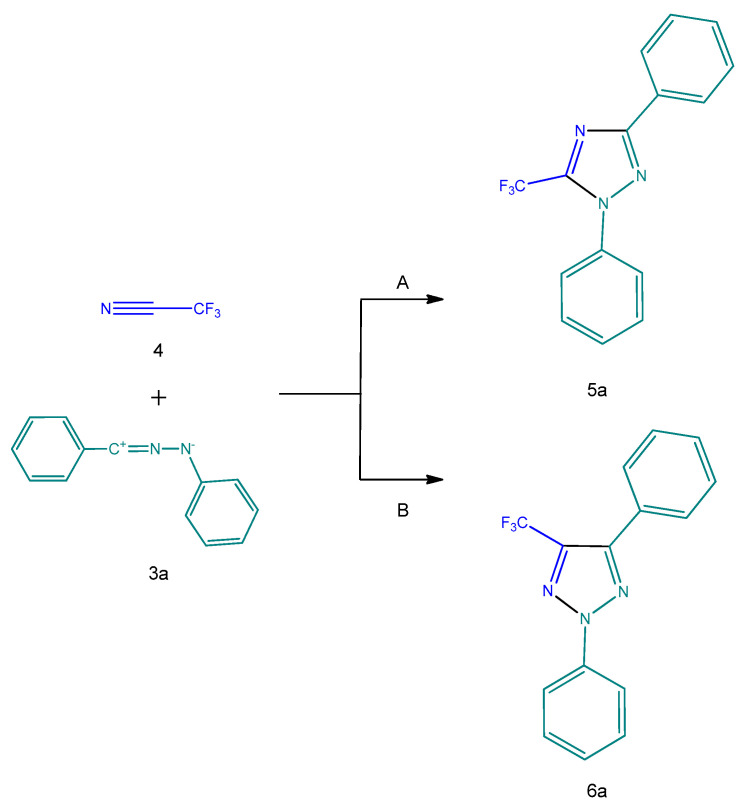
Theoretically possible regioisomeric 32CA reaction paths between NI and TFAN.

Due to the nonsymmetry of the reagents, the 32CA reaction of NI **3a**–**c** and TFAN **4** can take place along two feasible regioisomeric reaction paths (Scheme 5). While regioisomeric path A is associated with the most electronically favorable N1–C5 interaction, path B is associated with the formation of the C3–C5 bond. Stationary points corresponding to molecular complexes (MCs), transition complexes (TSs), and cycloadducts were located and characterized on both reaction paths. Only one TS was found in each pathway, indicating a one-step mechanism.

The quantum–chemical calculations show that the 32CA reaction of **3a**–**c** with **4** leads to MCs at the initial stage in the two reaction channels. MCs are created without the necessity of crossing an activation barrier. Since MCs are in the thermodynamic equilibrium, only the most stable one among the regioisomeric paths A and B was considered the energy reference. Further conversion of the MCs along the reaction paths leads to TSs, as confirmed by IRC analyses.

The experimental studies [33] indicate the formation of product 5, a finding corroborated by the calculations performed herein. Moreover, in the case of derivatives with a substituent in the aryl ring, this feature remains.

**Figure 5:**
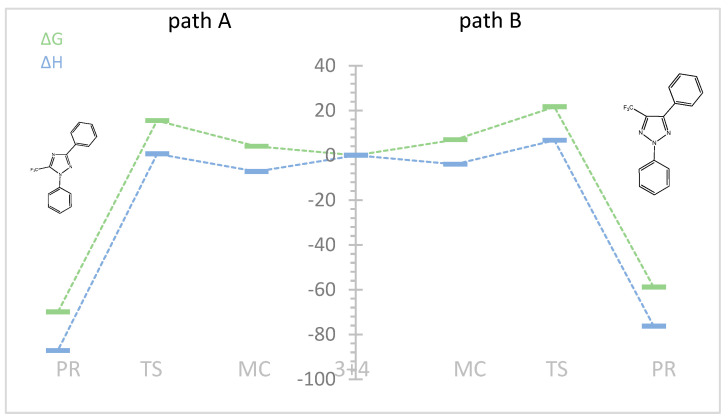
Energy profiles for paths A and B of the 32CA of NI (**3a**) and TFAN (**4**) according to ωB97X-D/6-311g(d,p) calculations.

Figure 5 presents energy profiles for paths A and B, while Table 3 summarizes the thermodynamic parameters of the stationary points involved in the 32CA reaction of NI with TFAN.

The reaction of TFAN **4** with NI **3** starts with the formation of the molecular complexes MC_5_ and MC_6_, which is associated with the growth of Gibbs free energy to 3.0–4.0 kcal mol^−1^ and 6.0–7.3 kcal mol^−1^, respectively. Further transformation leads to the transition states TS_5_ and TS_6_. The Gibbs free energy values for the TSs are 4.0 kcal mol^−1^ for TS_5a_ and 21.7 kcal mol^−1^ for TS_6a_. For NI **3a**, the activation Gibbs free energies associated with the regioisomeric path A via TS_5_ are 6.2 kcal mol^−1^ lower than those corresponding to path B via TS_6_. For **3b** and **3c**, these values are 4.6 and 5.3, respectively. These results indicate that the 32CA reaction between NI **3** and TFAN **4** is completely regioselective, leading to the formation of product **5** in complete agreement with the experimental findings [33]. From **3a** to **3c**, the substitution slightly decreases the activation of Gibbs free energies, probably due to the increased carbenoid character of **3c**.

The reaction of Gibbs free energies is ca. −70 kcal mol^−1^ for path A and −60 kcal mol^−1^ for path B. These highly negative values indicate that the processes are irreversible; therefore, the formation of **5** is exclusively under the kinetic control of the reaction.

**Figure 6:**
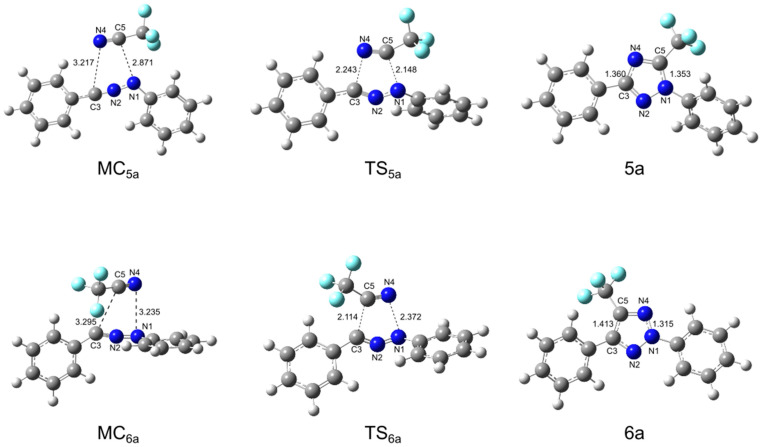
ωB97X-D/6−311g(d,p): Geometries of the critical structures in the [3+2] cycloaddition reaction of NI and TFAN.

The stationary points involved in the 32CA reaction between NI **3a** and TFAN **4** are shown in Figure 6. In MC_5a_, the N-C distances are considerably long and asymmetric, with the N1-C5 distance being shorter at ca. 2.87 Å than the distance between C3 and N4 at ca. 3.22 Å. The atoms in both the NI and TFAN frameworks maintain a linear geometry. In TS_5a_, the geometries of both frameworks are slightly bent, and a reduction in the bond-forming distances can be observed. The N1–C5 and C3–N4 atomic distances at TS_5a_ of 2.148 and 2.243 Å, respectively, are shorter compared to the initial MC_5a_ and are within the expected ranges typical of this type of structure. The N1–C5 bond is slightly shorter than the C3–N4 one, suggesting a more advanced bond formation of the N1–C5 bond. This finding is in agreement with the previous analysis of the Parr functions, showing the most favorable interaction between the most nucleophilic N1 nitrogen and the most electrophilic C5 carbon. Finally, at **5a**, the lengths of the C-N bonds are alike at 1.353 Å (N1–C5) and 1.360 Å (C3–N4).

To assess the asynchronicity of the studied 32CA reactions, an analysis was conducted on all stationary points. Key parameters, such as the interatomic distance between reaction centers (r), the distance progress index (l) and the asymmetry index Δl, are presented in Table 2. The distance progress indexes of the forming l_C3–N4_ and l_C5–N1_ are 0.35 and 0.41 for TS_5a_, respectively. These values indicate that for TS_5a_, the formation of the C5-N1 bond is more advanced than the C3–N4 bond. A similar situation is observed in the case of pathway B. For TS_6a_, the distance progress indexes of the forming l_N1–N4_ and l_C5–C3_ are 0.20 and 0.50, respectively. This information shows that while TS_5a_ is only slightly asynchronous, the unfavorable regioisomeric TS_6a_ presents a significant asynchronicity, with the formation of the N1–N4 bond being somewhat delayed. In addition, it can be concluded that the more favorable TS_5a_ is slightly earlier than TS_6a_.

Additionally, to confirm the polar nature of the 32CA reactions, the values of global electron density transfer (GEDT) [37] for the transition states (TSs) were calculated (Table 4). The GEDT values are 0.15e for TS_5a_ and 0.22e for TS_6a_, indicating a relatively polar process. The positive GEDT value shows the electron density flowing from NI to TFAN, supporting a forward electron density flux (FEDF) reaction where NI acts as the nucleophile in agreement with predictions from Conceptual Density Functional Theory (CDFT). The unfavorable regioisomeric TS_6a_ is slightly more polar than TS_5a_, but this is only a consequence of the more advanced character of the former. Note that GEDT not only depends on the global and local nucleophilic and electrophilic activation of the reagents but also the position of the TSs along the reaction path.

### 2.4. Bonding Evolution Theory (BET)

Finally, we performed a Bonding Evolution Theory (BET) [52] study for full comprehension of the molecular mechanism of the reaction between NI **3** and TFAN **4**. To achieve this, the most favorable reaction path A, associated with the simpler 32CA reaction between NI **3a** and TFAN **4,** was selected. This quantum−chemical methodology makes it possible to understand the bonding changes along a reaction path and, thus, to establish the nature of the electronic rearrangement associated with a given molecular mechanism [53].

**Figure 7:**
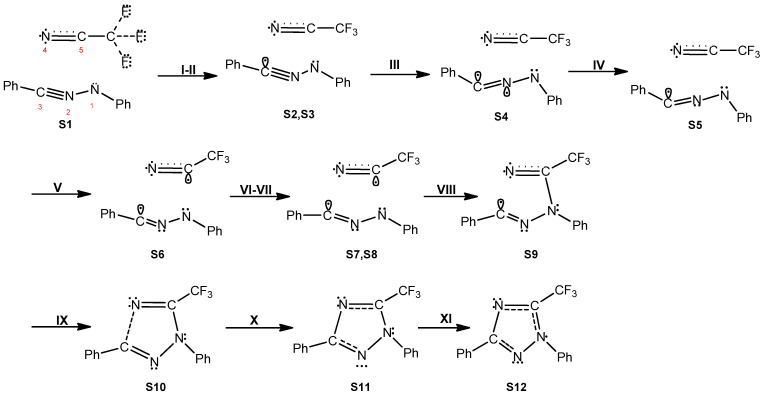
Simplified representation of the molecular mechanism of the reaction between NI **3a** and TFAN **4** using ELF−based Lewis structures.

The analysis of the ELF valence basin population changes along the IRC of the 32CA reaction between NI **3a** and TFAN **4** shows that this reaction can be topologically characterized by 11 differentiated phases (see Figure 7 and Table 5).

Phase I, 2.863Å ≥ d(N1−C5) > 2.845 Å and 3.182Å ≥ d(C3−N4) > 3.125 Å, starts at 01, which is the first point on the IRC path. The electronic structure of this system resembles that of the separated reagents (Figure 1 and Figure 2).

In Phase II, 2.845 Å ≥ d(N1−C5) > 2.753 Å and 3.125 Å ≥ d(C3−N4) > 2.939 Å, which starts at 051, a monosynaptic basin V (C3), integrating 0.28e, appears at the C3 carbon in the TAC moiety. This monosynaptic basin, associated with a C3 *pseudoradical* center, progressively increases its population. The population in the N2-C3 bonding region decreases.

In phase III, 2.753 Å ≥ d(N1−C5) > 2.426 Å and 2.939 Å ≥ d(C3−N4) > 2.531 Å, which starts at 096, the two disynaptic basins V (N2, C3) and V′ (N2, C3), integrating a total of 5.49e at the beginning of phase II, merge into a single disynaptic basin V (N1, N2) integrating 5.34e.

In Phase IV, 2.426 Å ≥ d(N1−C5) > 2.347 Å and 2.531 Å ≥ d(C3−N4) > 2.450 Å, the population of the V (N2, C3) disynaptic basin present in the last phase III decreases to 4.46e and keeps decreasing along the phase. This change is related to the creation of the double bond between N2 and C3 atoms, together with the appearance of a new V (N2) monosynaptic basin integrating 0.85e, which is associated with the N2 nitrogen non-bonding electron density.

In Phase V, 2.284 Å ≥ d(N1−C5) > 2.056 Å and 2.386 Å ≥ d(C3−N4) > 2.148 Å, this V (N2) monosynaptic basin reaches 1.81e. In addition, the V(N4, C5) disynaptic basin associated with the overpopulated double bond of the TFAN framework, integrating 4.49e in phase IV, splits into two V (N4, C5) and V′ (N4, C5) disynaptic basins with a total population of 4.44e. The transition state (TS_5a_, d(N1−C5) = 2.284 Å, and d(N3−C4) = 2.836 Å) of the reaction is found in this phase.

In Phase VI, 2.056 Å ≥ d(N1−C5) > 1.859 Å and 2.148 Å ≥ d(C3−N4) > 1.933 Å, a new V(C5) monosynaptic basin, integrating 0.25e, is created. This is associated with a C5 pseudoradical center.

In Phase VII, 1.859 Å ≥ d(N1−C5) > 1.851 Å and 1.933 Å ≥ d(C3−N4) > 1.924 Å, the V(C3) monosynaptic basin splits into two V (C3) and V′ (C3) monosynaptic basins, integrating 0.63e and 0.28e, respectively. This is only a consequence of the C3 carbon being in a planar geometrical environment. The population of the V (C5) monosynaptic basin increases to 0.55e.

In Phase VIII, 1.851 Å ≥ d(N1−C5) > 1.789 Å and 1.924 Å ≥ d(C3−N4) > 1.857 Å, the V(N1) monosynaptic basin, integrating 3.26e in previous phase VII, splits into two V(N1) and V’(N1) monosynaptic basins, integrating 2.18e and 1.09e, respectively. This is in preparation to form the N1-C5 bond in the next phase.

In Phase IX, 1.789 Å ≥ d(N1−C5) > 1.772 Å and 1.857 Å ≥ d(C3−N4) > 1.837 Å, the bond between N1 and C5 is created, since a new V (N1, C5) disynaptic basin appears with a population of 1.73e through the merger of both V (N1) and V (C5) monosynaptic basins. Thus, the formation of this bond takes place by sharing the non-bonding electron densities of the N1 and C5 atoms. In the formation of this bond, the N1 non-bonding electron density has a greater weight.

In Phase X, 1.772 Å ≥ d(N1−C5) > 1.634 Å and 1.837 Å ≥ d(C3−N4) > 1.683 Å, the V (C3) and V′ (C3) monosynaptic basins disappear, and a new V (C3, N4) disynaptic basin appears, integrating 1.05e. This change is accompanied by a significant depopulation of the V (N4) monosynaptic basin by 0.60e. These topological changes are related to the formation of the second C3−N4 single. In addition, the V (N4, C5) and V′ (N4, C5) disynaptic basins, integrating 3.55e at the beginning of Phase IX, merge into a new V (N4, C5) disynaptic basin integrating 3.34e.

Finally, in Phase XI, 1.634 Å ≥ d(N1−C5) ≥ 1.353 Å and 1.683 Å ≥ d(C3−N4) ≥ 1.360 Å, the population of the V (N1) monosynaptic basin decreases from 1.93e to 0.71e, and the populations of the V (N1, C5) and V (C3, N4) disynaptic basins increases to 3.03e and 2.20, respectively.

## 3. Computational Details

The DFT calculations were conducted employing the hybrid ωB97X−D functional [54], which incorporated a long-range exchange correction (designated by X), as well as semiclassical London dispersion correction (denoted by the suffix −D). The standard 6−311G(d,p) basis set [55] was utilized, encompassing d-type polarization for the second-row elements and p-type polarization functions for hydrogen atoms. Optimizations were performed using the Berny method [56,57]. This methodology is commonly applied in the investigation of the mechanistic aspects of the synthesis of heterocyclic compounds [58,59] and especially in the protocol of cycloaddition reactions [60,61,62,63]. The transition states (TSs) were characterized via frequency analysis, revealing only one imaginary frequency. The intrinsic reaction coordinate (IRC) paths [64] were determined. The essential parameters for the reactivity descriptors were computed using the following formulae:

The chemical potential [42,46,65,66,67]:

µ ≈ (ε_HOMO_ + ε_LUMO_)/2

The chemical hardness [64,65,67]:

η ≈ (ε_LUMO_ − ε_HOMO_)

The global electrophilicity index [42]:

ω = (µ^2^/2η)

The global nucleophilicity:

N = E_HOMO_(Nu) − E_HOMO_(TCE)

where tetracyanoethylene (TCE) is the reference.

The local electrophilicity was calculated based on global properties and the Parr function (P^+^_k_ or P^−^_k_) [51]:

ω_k_ = P^+^_k_·ω

The local nucleophilicity:

N_k_ = P^−^_k_·N

The topological analyses of the electron localization function (ELF) [39,68,69] were performed with TopMod 09 [70] software based on monodeterminantal wavefunctions over a grid with a spacing of 0.1 atomic units. For the BET [52] studies, the topological analysis of the ELF along the IRC was performed for a total of 498 nuclear configurations for the reaction of cycloaddition.

The global electron density transfer (GEDT) [71] values were determined via Natural Population Analysis (NPA) [72,73] using the expression GEDT(f) = charge qf, where q represents the atomic charges within the framework (f) at the transition states (TSs). The distance progress indices (l) were calculated according to the established formula [74]:

$$l_{X-Y}=1-\frac{r_{X-Y}^{TS}-r_{X-Y}^{P}}{r_{X-Y}^{P}}$$

where r^TS^_X−Y_ is the distance between the reaction centers X and Y in the transition structure, and r^P^_X−Y_ is the same distance in the corresponding product.

The calculations were carried out at the ωB97X-D/6-311g(d,p) level of theory in the gas phase. We conducted the calculations in the GAUSSIAN 16 package [75]. The visualization of molecular geometries, as well as 3D representations of radical anions, radical cations, and ELF basin attractors, were achieved using GaussView 6.0 [76] software. The ELF localization domains, set at an isovalue of 0.75 atomic units, were generated using Paraview 5.9.1 software for further spatial analysis. For the optimized structures, thermochemical data were derived for a temperature of T = 298 K and a pressure of p = 1 atm based on the vibrational analysis.

The quantum–chemical calculations reported in this paper were performed in the Ares computer cluster of the CYFRONET regional computer center in Cracow and the server of the MEDT research group at the University of Valencia.

## 4. Conclusions

In this study, a theoretical investigation of the 32CA reaction between CF_3_CN (TFAN **4**) and nitrilimine (NI **3**) was conducted using the MEDT framework. The reaction preferentially proceeded toward the formation of 1,3-diphenyl-5-trifluoromethyl-1,2,4-triazole (**5**) due to the most favorable interaction between the most nucleophilic N1 center of NI **3** and the most electrophilic C5 center of TFAN **4**, as shown by the analysis of the Parr functions. The alternative regioisomers of 2,4-diphenyl-5-(trifluoromethyl)-2*H*-1,2,3-triazole (**6**) are less favored, as evidenced by the experimental data where the formation of the second reaction product was not observed. The presence of substituents at the para-position on the phenyl ring of NIs (**3a**–**c**) does not significantly impact regioselectivity. According to Gibbs free energies, the reaction presents a total regioselectivity since TS_6_ is ca. 5 kcal/mol above TS5. Gibbs free activation energies are relatively low at ca. 15 kcal/mol and in complete agreement with the previous activation energies reported for 32CA reactions of carbenoid TACs [77]. The formation of regioisomer **5** is irreversible and favored by both the kinetic and thermodynamic control conditions of the reaction. Mechanistically, the formation of the N1–C5 bond takes place before the N3–C4 one by sharing the non-bonding electron densities of the nitrogen and pseudoradical carbon centers. The fact that both bonds form by sharing the non-bonding electron density of a nitrogen atom and a carbon *pseudoradical* center, which is present very early at the beginning of the reaction, suggests that NI **3a** behaves as a pseudo(mono)radical TAC in a polar pmr-type 32CA reaction of FEDF toward TFAN **4**. The inclusion of electron-donating groups at **3a** may transform **3a** into a carbenoid TAC participating in cb-type 32CA reactions, as previously shown in the 32CA reactions of substituted azomethine ylides [36]. This study shows the changing structure of NIs with substitutions.

## Figures and Tables

**Table 1:** ωb97X-D/6-311g(d,p): the most significant ELF valence basin populations N for molecules **3a–c**, given as average number of electrons [e].

	3a	3b	3c
ELF Basins	N [e]	N [e]	N [e]
V (N1, C1′)	1.91	1.92	1.93
V (N1, N2)	2.21	2.23	2.22
V (N2, C3)	2.43	2.42	2.23
V′ (N2, C3)	2.76	2.73	2.47
V (C3, C3′)	2.98	3.05	2.26
V (N1)	3.44	3.41	3.34
V (C3)			1.34

**Table 2:** ωB97X-D/6-311g(d,p). Electronic chemical potential (μ), chemical hardness (η), electrophilicity (ω) and nucleophilicity (N) in eV of NI and TFAN.

	Homo eV	Lumo eV	µ (eV)	ŋ (eV)	ω (eV)	N (eV)	Electrophile	Nucleophile
**3a**	−6.95	0.03	−3.46	6.98	0.86	4.44	moderate	superstrong
**3b**	−7.11	−0.60	−3.86	6.51	1.14	4.28	strong	superstrong
**3c**	−7.23	0.36	−3.43	7.59	0.78	4.17	moderate	superstrong
**4**	−13.06	1.19	−5.93	14.25	1.23	−1.66	strong	marginal

**Table 3:** ωB97X-D/6-311g(d,p). Relative enthalpies (ΔH in kcal·mol^−1^), entropies (ΔS in cal·mol^−1^ K^−1^) and Gibbs free energies (ΔG in kcal·mol^−1^) in gas phase of the stationary points involved in the 32CA reaction of NI (**3**) with TFAN (**4**). Relative energies are given with respect to the separated reagents NI **3a**–**c** and TFAN **4**.

Path	Stationary Point	ΔH	ΔS	ΔG
A	**MC_5a_**	−7.2	−37.6	4.0
	**TS_5a_**	0.7	−49.8	15.5
	**5a**	−87.1	−57.5	−70.0
	**MC_5b_**	−7.1	−34.1	3.0
	**TS_5b_**	1.2	−47.7	15.4
	**5b**	−87.3	−55.8	−70.6
	**MC_5c_**	−7.0	−73.2	3.2
	**TS_5c_**	0.4	−46.6	14.3
	**5c**	−86.4	−54.0	−70.3
B	**MC_6a_**	−4.0	−36.8	7.0
	**TS_6a_**	6.7	50.2	21.7
	**6a**	−76.3	−58.3	−58.9
	**MC_6b_**	−3.4	−35.7	7.3
	**TS_6b_**	6.0	−47.0	20.0
	**6b**	−76.4	−55.1	−59.9
	**MC_6c_**	−3.9	−33.5	6.0
	**TS_6c_**	5.7	−46.8	19.6
	**6c**	−75.6	−53.8	−59.5

**Table 4:** The key parameters of the critical structure parameters of reaction of NI and TFAN according to ωB97X-D/6-311g(d,p) calculations.

	**r_C3_** **_–_** **_N4_** **[Å]**	**l_C3_** **_–_** **_N4_**	**r_C5_** **_–_** **_N1_** **[Å]**	**l_C5_** **_–_** **_N1_**	**Δl**	**GEDT [e]**
**MC_5a_**	3.217		2.871			
**TS_5a_**	2.243	0.35	2.148	0.41	0.06	0.15
**5a**	1.361		1.353			
	**r_C5_** **_–C3_ [Å]**	**l_C5_** **_–C3_**	**r_N1_** **_–N4_ [Å]**	**l_N1_** **_–N4_**	**Δl**	**GEDT [e]**
**MC_6a_**	3.295		3.235			
**TS_6a_**	2.114	0.50	2.372	0.20	0.30	0.22
**6a**	1.413		1.315			

**Table 5:** Method ELF valence basin populations of the most relevant structures obtained from the IRC path along the most favorable regioisomeric reaction path A of the 32CA reaction between NI **3a** and TFAN **4**.

Structures	S1	S2	S3	S4	S5	S6	S7	S8	S9	S10	S11	S12
**Phases**	I	II	III	IV	V	VI	VII	VIII	IX	X	XI	
**d1 (N1** **−** **C5)**	2.863	2.845	2.753	2.426	2.284	2.056	1.859	1.851	1.789	1.772	1.634	1.353
**d2 (N3−C4)**	3.182	3.125	2.939	2.531	2.386	2.148	1.933	1.924	1.857	1.837	1.683	1.360
**V (N1, N2)**	2.11	2.10	2.08	1.94	1.86	1.75	1.67	1.67	1.65	1.64	1.61	1.61
**V (N2)**				0.85	1.81	2.18	2.47	2.48	2.56	2.58	2.73	3.05
**V (N2, C3)**	3.36	3.39	5.34	4.46	3.52	3.26	3.10	3.10	3.07	3.53	3.33	2.81
**V′ (N2, C3)**	2.36	2.10										
**V (C3, C3′)**	2.50	2.47	2.46	2.44	2.42	2.38	2.33	2.33	2.32	2.32	2.29	2.31
**V (N4, C5)**	4.56	4.56	4.56	4.49	2.15	2.07	1.89	1.88	1.84	1.83	3.34	2.84
**V′ (N4, C5)**					2.29	2.06	1.80	1.79	1.74	1.72		
**V (N1)**	3.52	3.53	3.51	3.45	3.42	3.34	3.26	2.18	2.12	2.10	1.93	0.71
**V′ (N1)**								1.09				
**V (C5)**						0.25	0.55	0.55				
**V (C3)**		0.28	0.45	0.66	0.73	0.85	0.63	0.62	0.40			
**V′ (C3)**							0.28	0.29	0.51			
**V (N4)**	3.13	3.13	3.14	3.20	3.26	3.39	3.58	3.59	3.65	3.05	2.94	2.88
**V (N1, C5)**									1.73	1.75	1.96	3.03
**V (C3, N4)**										1.05	1.46	2.20

## Data Availability

The data presented in this study are available on request from the corresponding author.
